# Refining reperfusion assessment with flat-panel detector CT: association with final infarct volume after endovascular thrombectomy

**DOI:** 10.1093/esj/aakag104

**Published:** 2026-09-01

**Authors:** Junpei Koge, Tetsuya Hashimoto, Kenichiro Suyama, Koji Tanaka, Kiyonori Kuwahara, Eiji Fujiwara, Daijiro Kojima, Tatsunori Mase, Shiho Tanaka, Saki Hyoudou, Syouta Naito, Katsuyoshi Ito, Yuichi Hirose, Shoji Matsumoto

**Affiliations:** Department of Comprehensive Strokology, Fujita Health University School of Medicine, Toyoake, Japan; Department of Comprehensive Strokology, Fujita Health University School of Medicine, Toyoake, Japan; Department of Neurosurgery, Fujita Health University School of Medicine, Toyoake, Japan; Department of Comprehensive Strokology, Fujita Health University School of Medicine, Toyoake, Japan; Department of Neurosurgery, Fujita Health University School of Medicine, Toyoake, Japan; Department of Neurosurgery, Fujita Health University School of Medicine, Toyoake, Japan; Department of Neurosurgery, Fujita Health University School of Medicine, Toyoake, Japan; Department of Neurosurgery, Fujita Health University School of Medicine, Toyoake, Japan; Department of Neurosurgery, Fujita Health University School of Medicine, Toyoake, Japan; Department of Radiology, Fujita Health University Hospital, Toyoake, Japan; Department of Radiology, Fujita Health University Hospital, Toyoake, Japan; Department of Radiology, Fujita Health University Hospital, Toyoake, Japan; Department of Neurosurgery, Fujita Health University School of Medicine, Toyoake, Japan; Department of Comprehensive Strokology, Fujita Health University School of Medicine, Toyoake, Japan

**Keywords:** angiography, reperfusion, stroke, thrombectomy

## Abstract

**Introduction:**

Distal occlusion tracker (DOT) signs on immediate post-interventional flat-panel detector CT (FDCT) can identify residual vessel occlusions underestimated by conventional angiography after endovascular thrombectomy (EVT). However, whether these FDCT-detected occlusions are associated with tissue-level outcomes remains unclear. We investigated whether FDCT-augmented reperfusion grading better reflects tissue imaging outcomes than operator-assessed reperfusion grading in patients with ischaemic stroke.

**Patients and methods:**

We retrospectively analysed EVT-treated patients with expanded thrombolysis in cerebral infarction (TICI) ≥ 2b who underwent post-interventional FDCT and follow-up MRI. DOT signs were defined as punctiform or tubular hyperdensities on FDCT. Final infarct volume (FIV) was segmented using follow-up DWI. Reperfusion status was categorised as incomplete (TICI 2b) or near-complete/complete (TICI 2c/3) based on FDCT-augmented TICI assessed by a core laboratory or operator-graded TICI.

**Results:**

Among 200 patients, the median FIV was 20.0 mL (IQR, 5.9–74.6), and DOT signs were identified in 86 (43.0%). Operator-graded TICI classified 61 patients (30.5%) as having incomplete reperfusion, compared with 72 patients (36.0%) by FDCT-augmented TICI; 17 patients were reclassified. Compared with near-complete/complete reperfusion, incomplete reperfusion by FDCT-augmented TICI was associated with larger FIV (β = 24.0 mL; 95% CI, 3.6–44.4), whereas operator-defined incomplete reperfusion was not (β = 12.2 mL; 95% CI, −9.5 to 33.9). The model incorporating FDCT-augmented TICI explained slightly more variance in FIV than the model incorporating operator-graded TICI (adjusted *R*^2^ 0.256 vs 0.239).

**Conclusion:**

FDCT-augmented reperfusion assessment may provide additional tissue-relevant information after EVT by identifying residual vessel occlusions associated with larger FIV.

## Graphical Abstract

**Graphical Abstract:**
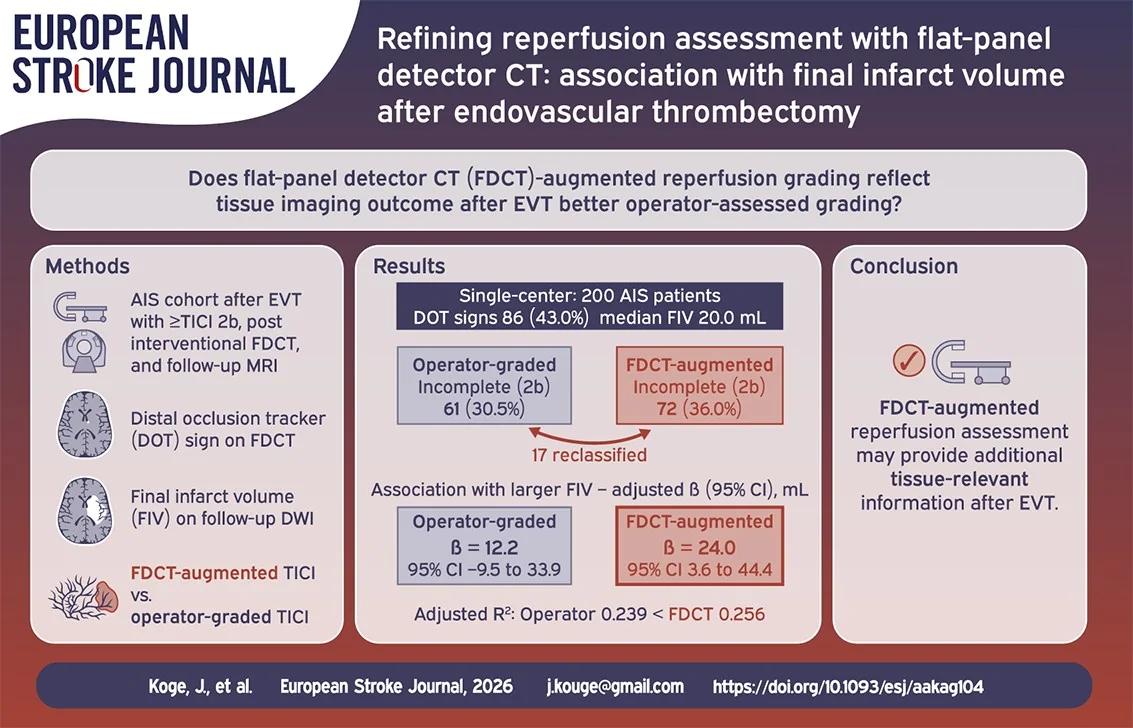

## Introduction

Endovascular thrombectomy (EVT) is the standard treatment for acute ischaemic stroke due to LVO.^1^^,^^2^ Reperfusion status after EVT is commonly assessed using the expanded thrombolysis in cerebral infarction (eTICI) scale.^3^ Although successful reperfusion is often defined as eTICI 2b or higher, accumulating evidence suggests that near-complete or complete reperfusion is associated with better outcomes than incomplete reperfusion.^4^ However, angiographic reperfusion grading is based on two-dimensional digital subtraction angiography (DSA) and relies on visual assessment of antegrade contrast filling, contributing to interobserver variability. Moreover, conventional angiographic grading may fail to capture residual distal vessel occlusions because of vessel overlap, collateral supply from adjacent territories or limited projectional information, particularly among patients classified as having successful reperfusion.^5^

This limitation of DSA-based reperfusion assessment is reflected in discordance between operator-assessed and core laboratory-adjudicated thrombolysis in cerebral infarction (TICI) grades.^6^ Such discordance is clinically relevant because overestimation of reperfusion may lead to premature termination of the procedure despite residual vessel occlusions that could be amenable to additional endovascular attempts or adjunctive therapies.^7^ Recently, the distal occlusion tracker (DOT) sign on immediate post-interventional flat-panel detector CT (FDCT) has been reported to help identify residual distal vessel occlusions.^8^ Incorporating FDCT findings into DSA interpretation has been shown to reduce missed distal occlusions and overestimation of reperfusion grades, even compared with core laboratory assessment based on DSA alone.^9^ However, whether residual vessel occlusions identified through FDCT-assisted detailed assessment are associated with tissue-level outcomes, particularly larger final infarct volume (FIV), remains unclear. Establishing this link is important because FIV represents a more direct imaging consequence of incomplete tissue reperfusion than early functional status alone.

We hypothesised that FDCT-augmented reperfusion assessment would identify residual vessel occlusions that were not captured by operator-assessed angiographic reperfusion grading and that these occlusions would be associated with larger FIV. This study aimed to investigate whether FDCT-augmented reperfusion grading better reflects tissue imaging outcomes after EVT than operator-assessed reperfusion grading in patients with acute ischaemic stroke.

## Patients and methods

### Study participants

This was a single-centre, observational cohort study. We retrospectively reviewed consecutive patients with acute ischaemic stroke who underwent EVT from January 2019 to May 2026. For patients who underwent more than 1 EVT during the same hospitalisation, only the first EVT procedure was considered. Patients were eligible if they met the following criteria: (1) patients achieving eTICI ≥ 2b at the end of the procedure; (2) availability of immediate post-interventional FDCT and (3) follow-up MRI performed within 5 days after EVT. Patients were excluded if (1) FDCT images were not evaluated due to artefacts or (2) 1-mm slice thickness FDCT reconstructions were not available. This study conforms to the Strengthening the Reporting of Observational Studies in Epidemiology (STROBE) guidelines for cohort studies. A completed STROBE checklist is included in the Online Supplementary Data.

### Standard protocol approvals, registrations and patient consents

The requirement for written informed consent was waived, and an opt-out approach was implemented because this retrospective study used anonymised data. Ethical approval was obtained from the local institutional review board.

### FDCT and follow-up MRI images acquisition

FDCT was routinely performed immediately after EVT, without additional contrast injection, to evaluate intracranial haemorrhage (ICH) or procedure-related complications. During the early study period, however, this protocol had not yet been fully standardised, and FDCT acquisition was partly left to the operator’s discretion. Thereafter, immediate post-interventional FDCT was routinely performed according to the institutional protocol. FDCT images were acquired using a biplane flat-panel detector angiographic system (Alphenix Biplane [INFX-8000V]; Canon Medical Systems, Otawara, Japan). Imaging parameters were as follows: voltage, 90 kV; frame speed, 30 frames/s; total angle, 216°; rotation speed, 10°/s; exposure time, 20 s; field of view, 12 inches and a 1024 × 1024 pixel matrix. Volume reconstruction was performed using a 1-mm slice thickness. Image reconstruction and post-processing were conducted using Alphenix Workstation Pro (Canon Medical Systems) or Ziostation REVORAS workstation (Ziosoft, Tokyo, Japan). Follow-up MRI images were obtained using 3 T or 1.5 T MRI systems from Canon Medical Systems (Vantage Titan, Vantage Galan and Vantage Centurian [3 T] and Vantage Orian [1.5 T]) and Philips Healthcare (Ingenia 3.0 T [3 T] and Achieva 1.5 T [1.5 T]; Best, the Netherlands). Follow-up MRI was performed within 24 ± 12 h after EVT as part of routine clinical practice. MRI obtained outside the above time window at the discretion of the treating physician was also included if performed within 5 days after EVT, consistent with a previous study.^10^

### Image analysis

All endovascular procedures were performed by neurointerventionalists certified by the Japanese Society for Neuroendovascular Therapy. The extent of baseline ischaemic change was graded using the Alberta Stroke Program Early CT Score (ASPECTS) on diffusion-weighted MRI or non-contrast CT. In patients with posterior circulation stroke, posterior circulation ASPECTS was assessed on diffusion-weighted MRI or on non-contrast CT or CTA source images. The occluded vessel was identified on baseline DSA. Reperfusion was graded on the final anteroposterior and lateral whole-brain DSA runs after EVT. Operator-graded TICI was assessed by the treating interventionalist at the end of the procedure according to the modified TICI scale, which included the 2c category but did not distinguish between 2b50 and 2b67, and was extracted from the interventional report.

The DOT sign was evaluated on FDCT acquired immediately after EVT. The DOT sign was considered present when a punctiform or tubular hyperdense signal increase was observed along the course of an intracranial artery in the ischaemic cerebral hemisphere. The number of DOT signs was also recorded and dichotomised as single or multiple. Baseline and follow-up imaging were reviewed to differentiate DOT signs from other hyperdense findings, including subarachnoid contrast extravasation and intracranial calcifications. The presence of DOT signs and FDCT-augmented TICI were assessed by a core laboratory (2 neurointerventionalists, each with > 10 years of experience) blinded to detailed clinical and procedural information.

FDCT-augmented TICI was assessed using final DSA and FDCT within the grading framework of the eTICI scale. DOT signs were used as adjunctive information to guide targeted review of the final DSA for residual vessel occlusions but did not automatically result in downgrading. The spatial location of each DOT sign was assessed on axial, coronal and sagittal FDCT reconstructions and correlated with the corresponding vascular location on the final DSA. Residual vessel occlusions were confirmed on the final DSA after correlation with the corresponding FDCT findings. Residual vessel occlusions identified on DSA despite the absence of corresponding DOT signs were also incorporated into FDCT-augmented TICI grading. The final grade was then assigned according to the extent of reperfusion on final DSA, taking into account the downstream territory of any confirmed residual occlusion. Throughout the manuscript, TICI^OP^ refers to operator-graded TICI, and TICI^FDCT^ refers to FDCT-augmented TICI. Incomplete reperfusion was defined as TICI 2b and near-complete/complete reperfusion as TICI 2c/3 on the final DSA run for both TICI^OP^ and TICI^FDCT^.

Final infarct lesions were segmented on follow-up DWI using dedicated software by a vascular neurologist with more than 15 years of experience, who was blinded to detailed clinical and procedural information. Infarcted areas in the affected cerebral hemisphere were manually delineated on multiple slices, with software-assisted interpolation between manually edited slices when appropriate. Corresponding apparent diffusion coefficient and fluid-attenuated inversion recovery (FLAIR) images were reviewed to help confirm infarcted tissue and exclude obvious artefacts or non-infarct signal abnormalities. The same reader subsequently reviewed the resulting segmentations and manually corrected them as needed. FIV was automatically calculated by the software based on the final multi-slice segmentation. Areas of haemorrhagic transformation within or adjacent to the infarcted territory were included in the infarct volume segmentation, whereas remote haemorrhages were not included, in accordance with prior core laboratory imaging outcome analyses.^11^^,^^12^ Lesion segmentation was performed on DWI rather than FLAIR at 5 days to avoid overestimation of FIV, consistent with a previous study.^13^ Zio software version 5.2.1.1 (Ziosoft, Tokyo, Japan) was used for segmentation and volumetric measurement.

### Clinical data collection

The following clinical variables were collected: age, sex, premorbid mRS score, baseline NIHSS score, cardiovascular risk factors (hypertension, diabetes mellitus, dyslipidaemia), congestive heart failure, atrial fibrillation, history of stroke or TIA and prior use of antiplatelet or anticoagulant agents.

### Outcome measures

The primary outcome was FIV (mL). Safety outcomes included any types of ICH within 36 h, classified according to the Heidelberg Bleeding Classification.^14^ Routine follow-up CT was performed within 36 h, or earlier in cases of clinical deterioration. ICH was primarily evaluated on follow-up CT. Haemorrhagic infarction (HI) was defined as HI1 or HI2, and parenchymal haematoma (PH) as PH1 or PH2. Any haemorrhagic transformation was defined as the presence of either HI or PH. Symptomatic ICH was defined as ICH associated with a ≥ 4-point increase in NIHSS score.^15^ Clinical outcomes were categorised as favourable outcome (mRS 0–2 or return to premorbid mRS at discharge), excellent outcome (mRS 0–1 or return to the premorbid mRS at discharge), mortality (mRS 6 at discharge) and shift in mRS score at discharge.

### Statistical analysis

Continuous data were summarised as medians with IQRs. Categorical data were presented as frequencies and percentages. Groups were compared using the Wilcoxon rank-sum test, Kruskal–Wallis test, Fisher’s exact test or Pearson’s chi-square test, as appropriate. Trends in the association between TICI grades and DOT signs were evaluated using the Cochran–Armitage test for trend. The association between TICI grades and FIV was assessed using Spearman’s rank correlation. Interrater agreement for the presence of the DOT sign was assessed using Cohen’s κ coefficient.

Multivariable linear regression models were constructed to evaluate the association between incomplete reperfusion defined by TICI^OP^ or TICI^FDCT^ and FIV. The following covariates were selected based on prior literature and clinical relevance: age, sex, baseline NIHSS score, ASPECTS, occlusion site categorised as internal carotid artery, M1, M2/3 segment of the middle cerebral artery, or posterior circulation, time from puncture to reperfusion (to eTICI ≥ 2b) and number of thrombectomy passes.^10^^,^^16–18^ The time from the end of EVT to follow-up MRI was included as an additional covariate to account for potential variability in FIV measurement related to MRI timing. For each model, results were presented as unstandardised regression coefficients (β) with 95% CIs. Model fit was compared using the adjusted *R*^2^, Akaike information criterion (AIC) and Bayesian information criterion (BIC). Differences in model fit indices between the TICI^OP^ and TICI^FDCT^ models were estimated with 95% CIs using 2,000 bootstrap resamples. As a descriptive analysis, standardised regression coefficients were also calculated to compare the relative contribution of each predictor to FIV. In sensitivity analyses, multivariable linear regression models were constructed in the subgroup of patients who underwent follow-up MRI within 48 h after EVT.

Multivariable logistic regression models were constructed for each binary outcome, and odds ratios (ORs) with 95% CIs were calculated. For safety outcomes, the following covariates were forced into the models: age, sex, baseline NIHSS score, ASPECTS, occlusion site, intravenous thrombolysis and the number of thrombectomy passes. For clinical outcomes, the same covariates used in the multivariable linear regression models were forced into the models, except for time from the end of EVT to follow-up MRI. Missing data were handled using pairwise deletion. A 2-sided *P* value < .05 was considered statistically significant. Statistical analyses were performed using R software (version 4.1.1; R Foundation for Statistical Computing, Vienna, Austria).

## Results

### Study population

The study flowchart is shown in Figure S1. Patient characteristics of the included cohort are summarised in Table 1. Baseline and procedural characteristics of included patients, patients without post-interventional FDCT and other excluded patients are presented in Table S1. The groups were generally comparable, although ASPECTS differed among the 3 groups and numerically lower in other excluded patients. A total of 200 patients (75 women [38%]; median age, 78 years [IQR, 72–84 years]; median baseline NIHSS score, 17 [IQR, 11–23]) were analysed. The DOT sign on FDCT was identified in 86 patients (43.0%). Interrater agreement for DOT sign identification was excellent (κ = 0.91; 95% CI, 0.84–0.96). Multiple DOT signs were observed in 38 patients (19.0%). Follow-up MRI was performed at a median of 21 h (IQR, 16–34 h) after the end of EVT, and the distribution of imaging timing is shown in Figure S2. The median FIV was 20.0 mL (IQR, 5.9–74.6).

**Table 1:** Patient characteristics.

	Overall (*n* = 200)
**Age, y**	78 [72–84]
**Females**	75 (37.5)
**Premorbid mRS score**	0 [0–2]
**Baseline NIHSS score^a^**	17 [11–23]
**ASPECTS^b^**	8 [6–9]
**Medical history**	
**Hypertension**	123 (61.5)
**Diabetes mellitus**	45 (22.5)
**Dyslipidaemia**	81 (40.5)
**Congestive heart failure**	25 (12.5)
**Atrial fibrillation**	114 (57.0)
**Previous stroke/TIA**	51 (25.5)
**Prior oral anticoagulants**	33 (16.5)
**Prior antiplatelets**	53 (26.5)
**Initial site of occlusion**	
**ICA**	65 (32.5)
**M1**	78 (39.0)
**M2/M3**	37 (18.5)
**Posterior circulation**	20 (10.0)
**First-line EVT strategy**	
**SR**	11 (5.5)
**CA**	107 (53.5)
**Combined SR and CA**	79 (39.5)
**Other**	3 (1.5)
**Intravenous thrombolysis**	84 (42.0)
**Number of thrombectomy passes**	1 [1–3]
**Onset-to-puncture time, min^c^**	264 [146–478]
**Puncture-to-eTICI ≥ 2b reperfusion time, min**	37 [24–61]
**Time from end of EVT to FDCT, min**	3 [2–8]
**Time from end of EVT to follow-up MRI, h**	21 [16–34]
**DOT sign**	
**None**	114 (57.0)
**Single**	48 (24.0)
**Multiple**	38 (19.0)
**Number of DOT signs**	0 [0–1]

Abbreviations: ASPECTS = Alberta Stroke Program Early CT score; ICA = internal carotid artery; M1 = first segment of the middle cerebral artery; M2 = second segment of the middle cerebral artery; M3 = third segment of the middle cerebral artery; EVT = endovascular thrombectomy; SR = stent retriever; CA = contact aspiration; eTICI = expanded thrombolysis in cerebral infarction; FDCT = flat-panel detector CT; DOT = distal occlusion tracker.

Data are presented as median [IQR] or number (%).

a Four missing values.

b ASPECTS was obtained using MRI (*n* = 88) or CT (*n* = 112).

c Nine missing values.

### Operator-graded TICI vs FDCT-augmented TICI

**Figure 1:**
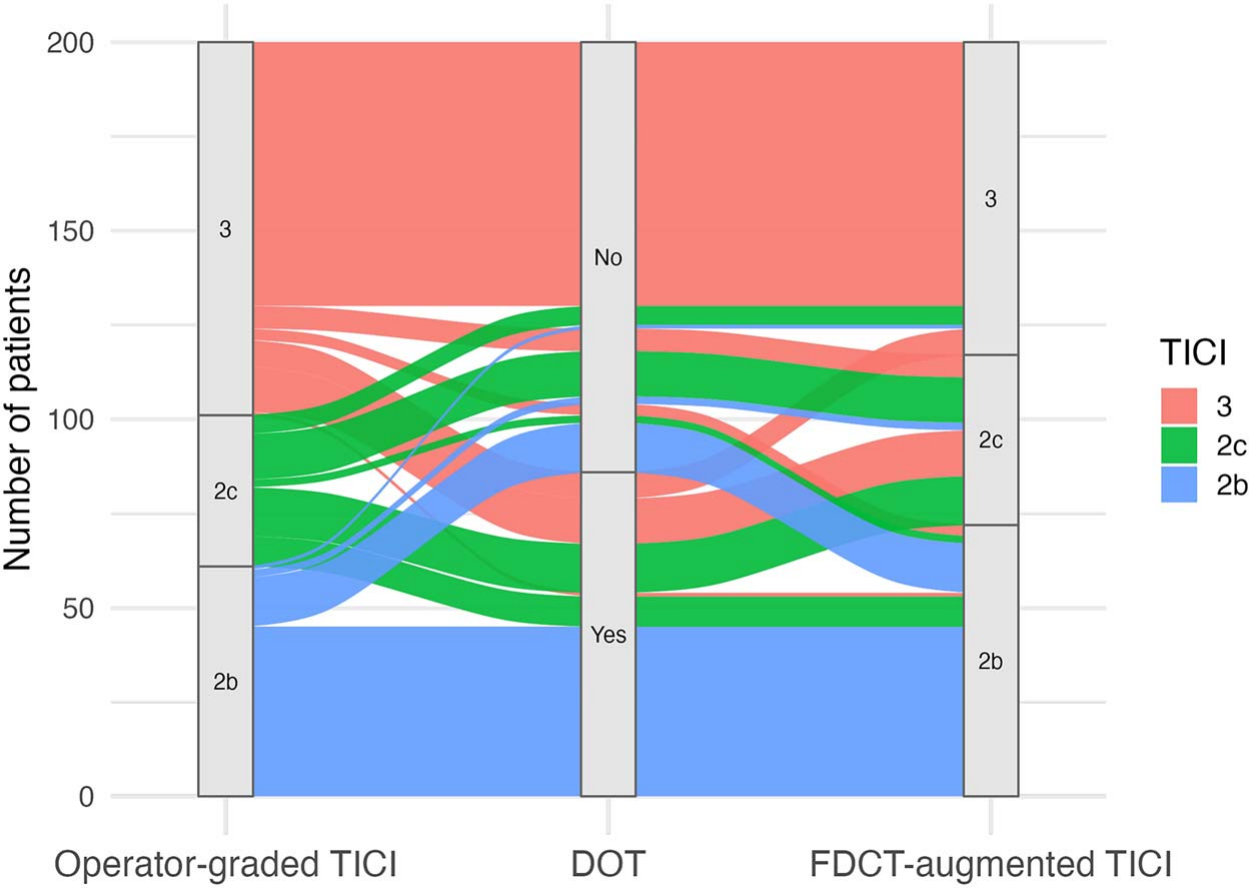
Alluvial diagram illustrating patient transitions across TICI categories between operator-graded TICI and FDCT-augmented TICI stratified by the presence of DOT signs. Abbreviations: DOT = distal occlusion tracker; FDCT = flat-panel detector CT; TICI = thrombolysis in cerebral infarction.

**Figure 2:**
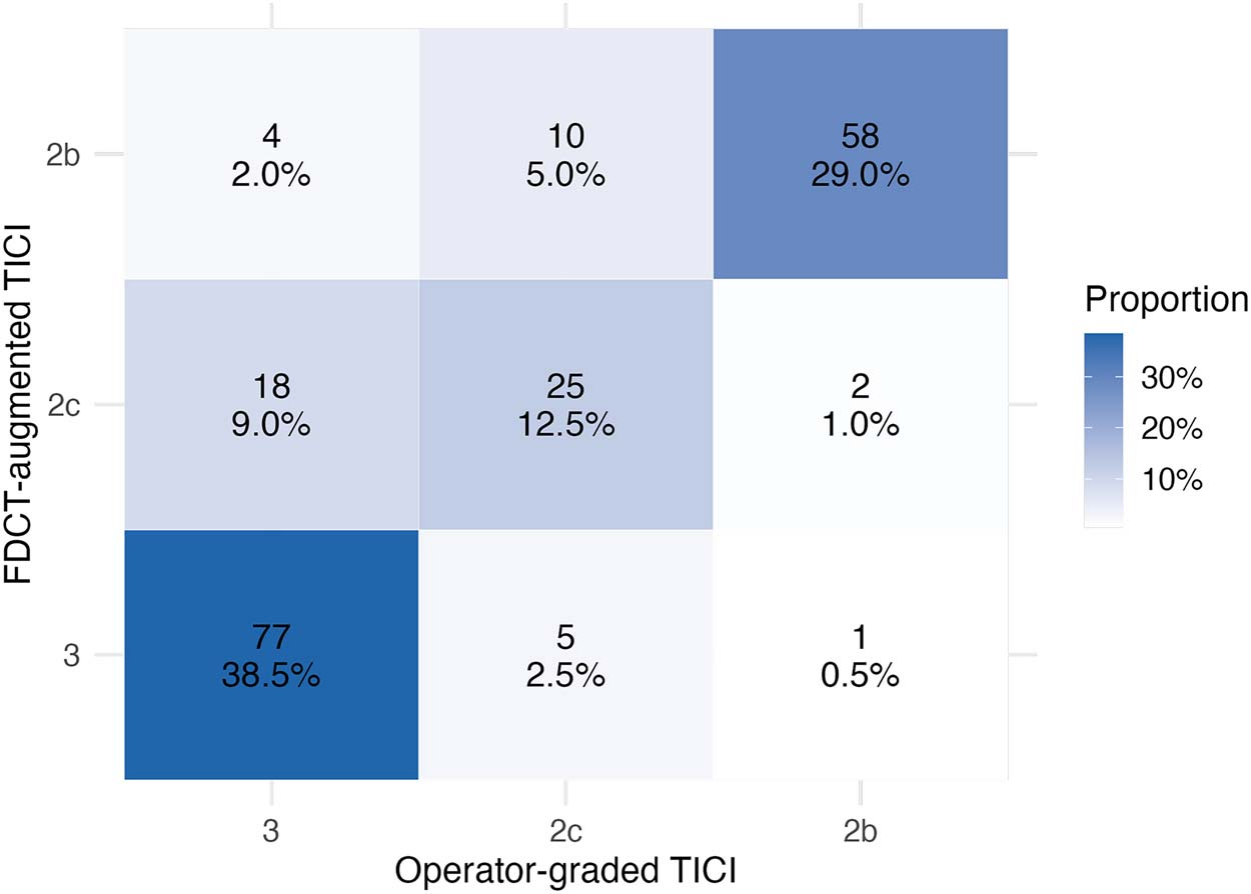
Heatmap illustrating the correlation between operator-graded TICI and FDCT-augmented TICI. Abbreviations: FDCT = flat-panel detector CT; TICI = thrombolysis in cerebral infarction.

Using TICI^OP^, 61 patients (30.5%) were classified as having incomplete reperfusion and 139 patients (69.5%) as having near-complete/complete reperfusion. According to TICI^FDCT^, incomplete reperfusion was observed in 72 patients (36.0%) and near-complete/complete reperfusion in 128 patients (64.0%). Among cases classified as near-complete/complete reperfusion by TICI^OP^, 14 were downgraded as incomplete by TICI^FDCT^; conversely, 3 cases classified as incomplete by TICI^OP^ were upgraded as near-complete/complete by TICI^FDCT^. Clinical and imaging characteristics of the 17 patients reclassified by TICI^FDCT^ are summarised descriptively in Table S2. An alluvial diagram illustrates patient transitions across TICI categories between TICI^OP^ and TICI^FDCT^, stratified by the presence of the DOT sign (Figure 1). Incorporating DOT signs demonstrated that a subset of cases initially classified as TICI^OP^ 3 were downgraded, whereas a subset of cases classified as TICI^OP^ 2c were reclassified as either TICI^FDCT^ 3 or 2b. A heatmap illustrates the correlation between TICI^OP^ and TICI^FDCT^, suggesting that TICI^OP^ tends to overestimate TICI 2c or TICI 3 compared with TICI^FDCT^ (Figure 2). A representative case classified as TICI 2c by TICI^OP^ but as TICI 2b by TICI^FDCT^ is shown in Figure S3. Patients with lower TICI grades on both TICI^OP^ and TICI^FDCT^ were more likely to exhibit DOT signs (both *P* < .001; Figure S4).

### Patient characteristics stratified by reperfusion status

Patient characteristics stratified by incomplete and near-complete/complete reperfusion according to TICI^FDCT^ are summarised in Table S3. Patients with incomplete reperfusion required a higher number of thrombectomy passes than those with near-complete/complete reperfusion. DOT signs were more frequently observed in patients with incomplete reperfusion than in those with near-complete/complete reperfusion. No significant differences were observed in other baseline or interventional characteristics or in workflow time metrics between groups. Patient characteristics stratified by incomplete and near-complete/complete reperfusion according to TICI^OP^ are shown in Table S4.

### Imaging outcomes

**Figure 3:**
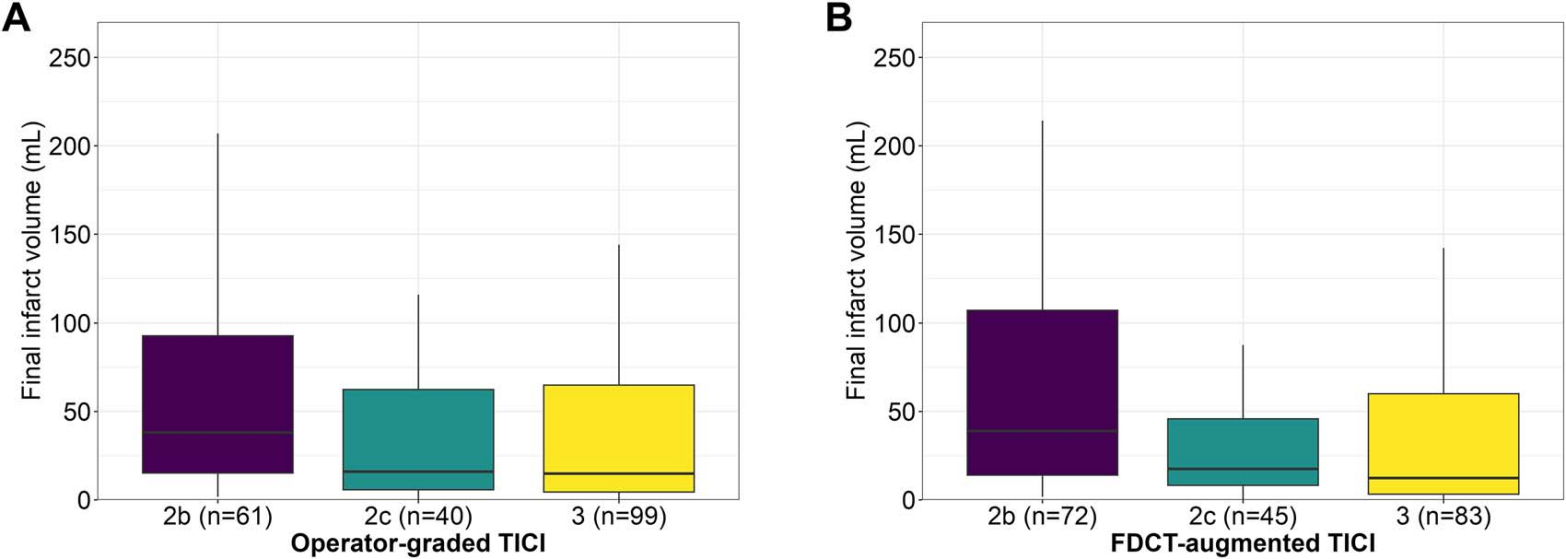
Boxplots of final infarct volumes by TICI grade based on operator-graded TICI (A) and FDCT-augmented TICI (B). Abbreviations: FDCT = flat-panel detector CT; TICI = thrombolysis in cerebral infarction.

**Figure 4:**
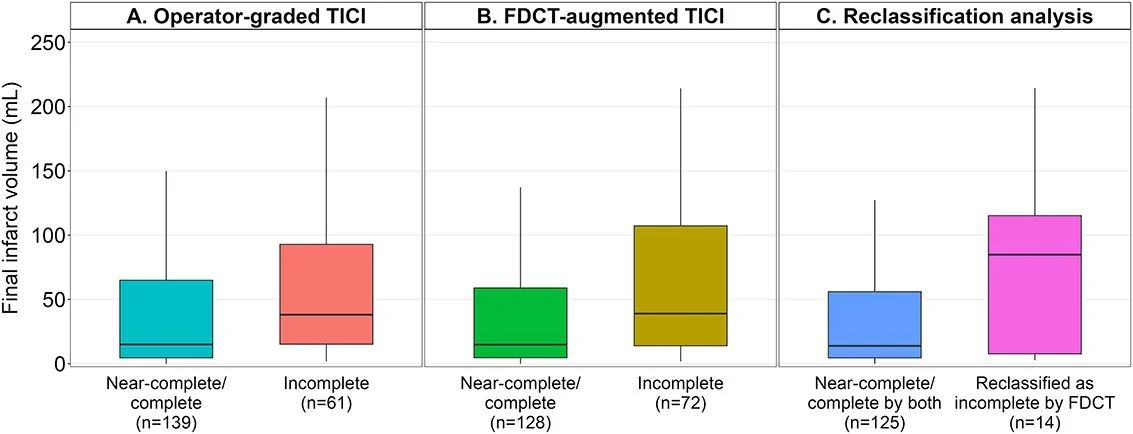
Final infarct volumes across reperfusion categories defined by operator-graded (A), FDCT-augmented TICI (B), and reclassification among patients with operator-graded near-complete/complete reperfusion (C). Abbreviations: FDCT = flat-panel detector CT; TICI = thrombolysis in cerebral infarction.

FIV across reperfusion groups is illustrated in Figure 3. For both TICI^OP^ (*P* = .002) and TICI^FDCT^ (*P* < .001), lower TICI grades were associated with larger FIV. Among patients with TICI 2c/3, patients with DOT signs tended to have larger FIV than those without. In contrast, patients with TICI 2b had relatively large FIV even without DOT signs (Figure S5). FIV was higher in patients with incomplete reperfusion than in those with near-complete/complete reperfusion when reperfusion was categorised using either TICI^OP^ (*P* = .001) or TICI^FDCT^ (*P* < .001) (Figure 4A, B). Among patients classified as having near-complete/complete reperfusion by TICI^OP^, the median FIV was higher in those reclassified as having incomplete reperfusion by TICI^FDCT^ than in those classified as having near-complete/complete reperfusion by both assessments (84.8 mL [IQR, 7.7–115.0] vs 13.8 mL [IQR, 4.5–56.0]; *P* = .04; Figure 4C). Among patients with near-complete/complete reperfusion by TICI^OP^, FIV was further stratified by the presence of DOT signs within patients who remained classified as near-complete/complete by TICI^FDCT^ and those reclassified as having incomplete reperfusion by TICI^FDCT^ (Figure S6). After adjustment for covariates, patients with incomplete reperfusion defined by TICI^FDCT^ had larger FIV than those with near-complete/complete reperfusion (β = 24.0 mL; 95% CI, 3.6–44.4). In contrast, patients with incomplete reperfusion defined by TICI^OP^ had numerically larger FIV than those with near-complete/complete reperfusion, although the difference did not reach statistical significance (β = 12.2 mL; 95% CI, −9.5 to 33.9) (Table 2). The TICI^FDCT^ model showed numerically more favourable model fit indices for FIV than the TICI^OP^ model, with lower AIC and BIC values and higher adjusted *R*^2^. However, differences were modest, and bootstrap 95% CIs crossed zero (Table 2). Sensitivity analyses yielded similar results (Table S5). A forest plot showing unstandardised regression coefficients for predictors of FIV in multivariable linear regression models is shown in Figure S7. Standardised coefficients for the 2 models are shown in Figure S8. The regression coefficients for incomplete reperfusion were larger in the TICI^FDCT^ model than in the TICI^OP^ model.

**Table 2:** Model performance of multivariable linear regression models either operator-graded TICI or FDCT-augmented TICI for predicting final infarct volume.

	Operator-graded TICI	FDCT-augmented TICI
**Incomplete reperfusion (TICI 2b), β coefficient (95% CI)**	12.2 (−9.5–33.9)	24.0 (3.6–44.4)
**Model performance**		
**Adjusted *R*^2^**	0.239	0.256
**AIC**	2,211.0	2,206.6
**BIC**	2,253.6	2,249.2
**Model difference favouring FDCT-augmented TICI**		
**ΔAdjusted *R*^2^**	0.017 (−0.003–0.049)	
**ΔAIC**	4.377 (−0.669–13.166)	
**ΔBIC**	4.377 (−0.669–13.166)	

Abbreviations: TICI = thrombolysis in cerebral infarction; FDCT = flat-panel detector CT; AIC = Akaike information criterion; BIC = Bayesian information criterion; ASPECTS = Alberta Stroke Program Early CT score; eTICI = expanded thrombolysis in cerebral infarction.

Adjusted for age, sex, baseline NIHSS score, ASPECTS, occlusion site, time from puncture to reperfusion to eTICI ≥ 2b, the number of passes, and time from the end of endovascular thrombectomy to follow-up MRI.

### Safety and clinical outcomes

Safety and clinical outcomes based on reperfusion status defined by TICI^FDCT^ are presented in Table S6. Patients with incomplete reperfusion defined by TICI^FDCT^ tended to have higher rates of PH than those with near-complete/complete reperfusion. The rate of subarachnoid haemorrhage was significantly higher in patients with incomplete reperfusion than in those with near-complete/complete reperfusion (adjusted OR [aOR], 4.05; 95% CI, 1.31–12.51). Incomplete reperfusion was associated with lower odds of favourable outcome (aOR, 0.35; 95% CI, 0.15–0.80) and excellent outcome (aOR, 0.40; 95% CI, 0.16–0.99).

Safety and clinical outcomes based on reperfusion status defined by TICI^OP^ are shown in Table S7. No significant differences were observed in any types of haemorrhagic transformation including PH, between incomplete and near-complete/complete reperfusion. Patients with incomplete reperfusion had a lower rate of favourable outcome (aOR, 0.38; 95% CI, 0.16–0.93) and tended to have a lower rate of excellent outcome than those with near-complete/complete reperfusion.

## Discussion

The major findings of the present study are as follows. First, incomplete reperfusion assessed by TICI^FDCT^ was significantly associated with larger FIV compared with near-complete/complete reperfusion, whereas incomplete reperfusion defined by TICI^OP^ was not. Second, the model incorporating incomplete reperfusion defined by TICI^FDCT^ demonstrated numerically more favourable model fit indices for FIV than the model incorporating TICI^OP^, although the magnitude of improvement was modest.

### DOT sign as an adjunct to reperfusion evaluation

The detection rate of DOT signs in the present study was lower than that reported by Mujanovic et al.,^8^ but comparable to that observed by Baik et al.,^9^ which similarly focused on patients achieving TICI ≥ 2b. In our cohort, the incorporation of DOT signs resulted in downgrading of both TICI^OP^ 3 and TICI^OP^ 2c, leading to a higher proportion of cases categorised as TICI 2b under TICI^FDCT^. Notably, among patients classified as TICI 2c, FIV estimates showed less apparent variability under TICI^FDCT^, as reflected by a narrower 95% CI compared with TICI^OP^. Our study extends existing evidence on the utility of DOT signs for reclassifying patients initially graded as having complete reperfusion^8^^,^^19^ by demonstrating that DOT-sign guided assessment can also identify tissue-relevant incomplete reperfusion (TICI 2b) among patients otherwise classified as having near-complete reperfusion (TICI 2c). Importantly, DOT signs can be assessed without additional contrast administration or specialised post-processing, and may therefore serve as a simple adjunct to reperfusion evaluation. However, the absence of DOT signs should not be interpreted as the absence of residual occlusion or tissue-level reperfusion impairment, because the DOT sign is highly specific but only moderately sensitive for residual vessel occlusion.^8^ In the present study, TICI 2b cases without DOT signs might therefore represent false-negative DOT findings, particularly in anatomical configurations where the short vessel segment between the residual thrombus and the adjacent proximal bifurcation provides insufficient space for contrast stagnation.^8^ Such residual occlusions may still affect multiple downstream vessels, which could partly explain larger FIV despite the absence of DOT signs.

### Association between FDCT-augmented TICI and FIV

The model incorporating TICI^FDCT^ demonstrated numerically more favourable model fit indices than that incorporating TICI^OP^, although the magnitude of improvement was modest and should be interpreted as supportive rather than definitive. Our findings extend prior work on the limitations of conventional angiographic reperfusion grading. Zhang et al. reported substantial disagreement between operator- and core laboratory-adjudicated modified TICI scores, with operators tending to overestimate reperfusion, although both assessments showed similar discrimination for 90-day functional outcome.^6^ In parallel, recent studies demonstrated that tissue-level hypoperfusion and infarct growth can occur even after near-complete or complete reperfusion.^7^^,^^20^ Taken together, these findings suggest that angiographic reperfusion grading, which primarily reflects vessel patency and downstream contrast opacification, may be relatively robust for broad clinical outcome prediction, but less sensitive to imaging outcomes that directly reflect tissue injury. By linking FDCT-augmented assessment of residual distal vessel occlusion with MRI-based tissue outcome, the present study suggests that TICI^FDCT^ may provide additional tissue-relevant information beyond angiography alone.

### Clinical relevance of larger FIV after incomplete reperfusion

In the present study, patients with incomplete reperfusion defined by TICI^FDCT^ had higher FIV than near-complete/complete reperfusion after adjustment for factors related to FIV and infarct growth.^10^^,^^16–18^ The difference in infarct volume between incomplete and near-complete/complete reperfusion in our cohort was larger than the infarct size difference previously reported between conventional TICI 2b and TICI 3 categories (26.11 mL vs 12.84 mL).^21^ A prior report suggested that a 20-mL difference in DWI-positive volume at 48 h corresponds to an approximately 10% difference in favourable outcome rates.^22^ Although functional outcome analyses in the present study were exploratory and should therefore be interpreted with caution, the magnitude of the observed difference in FIV was comparable to that previously associated with clinically meaningful differences in functional outcome. These findings suggest that the tissue-level differences identified by TICI^FDCT^ may also be clinically relevant.

Importantly, patients reclassified as having incomplete reperfusion by TICI^FDCT^ had larger FIV than those who remained classified as having near-complete/complete reperfusion by both TICI^OP^ and TICI^FDCT^. Although this observation should be interpreted as hypothesis-generating because of the limited number of reclassified patients, it suggests that FDCT-augmented assessment may help identify a subgroup of patients at risk for greater tissue injury despite apparently successful EVT. Such patients may represent a potential target population for closer post-procedural monitoring or future studies of adjunctive post-intervention therapies. However, the trade-off between additional radiation exposure from FDCT and the potential clinical benefit of detecting DOT signs remains unknown. Although recognition of DOT signs may prompt consideration of additional therapeutic measures, such as secondary distal thrombectomy, intra-arterial thrombolysis or antithrombotic therapy, whether such additional measures reduce FIV or improve clinical outcomes has yet to be demonstrated.

### Exploratory safety findings

In exploratory analyses, incomplete reperfusion defined by TICI^FDCT^ showed a trend towards an increased risk of PH. However, this finding should be interpreted cautiously because of the small number of events and the wide CI. A higher number of passes^23^ and impaired collateral circulation,^24^ potentially related to multiple distal vessel occlusions, may partly contribute to this trend, although these mechanisms remain speculative and causal interpretation cannot be made from the present data.

### Limitations

The present study has several limitations. First, the single-centre, retrospective design and relatively small sample size introduce inherent risks of bias and limit generalisability. In addition, baseline ASPECTS differed among included and excluded patients, suggesting that selection bias related to baseline infarct size cannot be excluded. Second, FIV may be influenced by the timing of follow-up MRI, because infarct volume can evolve over time after reperfusion.^25^^,^^26^ Although the median interval from the end of the intervention to follow-up MRI was approximately 21 h, follow-up timing varied among patients, and early imaging may have underestimated FIV in some patients, particularly in those with delayed infarct evolution beyond the 24-h period.^27^ Although MRI timing was included as a covariate in the multivariable models, residual effects of variable imaging timing cannot be excluded. Third, FIV was assessed on early follow-up MRI, with haemorrhagic areas within the ischaemic lesion included in the segmentation. Susceptibility-weighted imaging and later follow-up imaging were not systematically available; therefore, haemorrhage, oedema or susceptibility-related artefacts may have affected FIV estimates. Fourth, baseline infarct volume data were not available, precluding direct estimation of infarct growth, because baseline ischaemic change was assessed using non-contrast CT in more than half of the patients. Fifth, the spatial relationship between DOT signs and the infarct was not evaluated. Sixth, functional outcomes were assessed at the time of hospital discharge, which may introduce variability in the timing of outcome assessment. Seventh, unmeasured confounders including baseline infarct volume, collateral status, and post-procedural management, may have influenced FIV.

## Conclusion

Patients with incomplete reperfusion defined by TICI^FDCT^ had larger infarct volumes than those with near-complete/complete reperfusion. Compared with TICI^OP^, TICI^FDCT^ showed numerically more favourable model fit indices for FIV, suggesting that adjunctive FDCT may provide additional tissue-relevant information for reperfusion assessment after EVT.

## Supplementary Material

Supplemental_material_ver7_R1_aakag104

## Data Availability

This study’s data are available from the corresponding author upon reasonable request.
