# Supplementary material for: Refining reperfusion assessment with flat-panel detector CT: association with final infarct volume after endovascular thrombectomy
Source: Eur Stroke J. 2026 Sep 1;11(8):aakag104. doi: 10.1093/esj/aakag104 (PMC13532578; doi:10.1093/esj/aakag104)
Supplement: Supplementary file 1 [file supplemental_material_ver7_r1_aakag104.pdf]

SUPPLEMENTAL MATERIAL

Refining reperfusion assessment with flat-panel detector CT: association with final infarct volume after endovascular thrombectomy

Figure 1. Study flowchart.

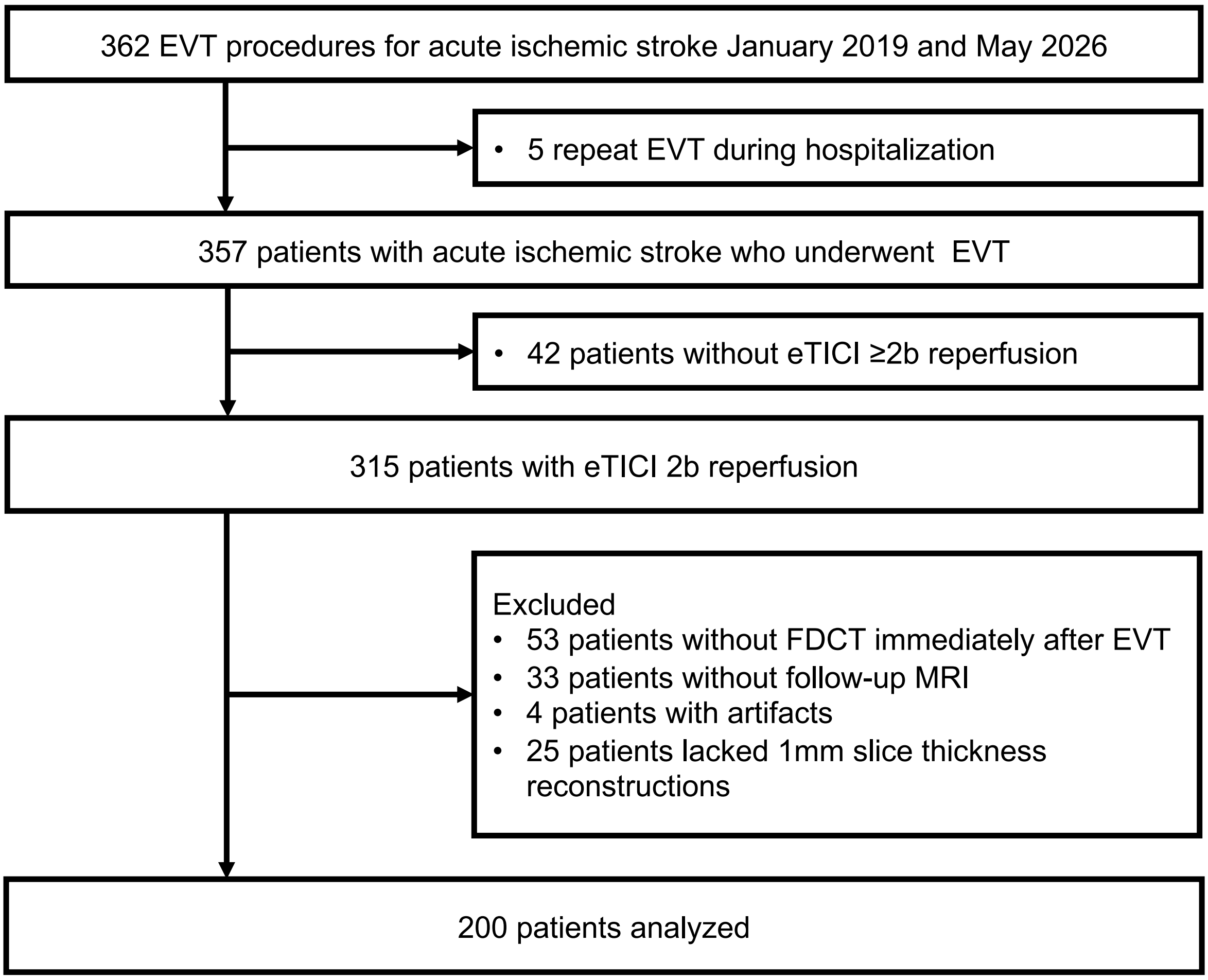

Abbreviations: EVT, endovascular thrombectomy; eTICI, expanded Thrombolysis In Cerebral Infarction; FDCT, flat-panel detector computed tomography; MRI, magnetic resonance imaging.

Figure 2. Distribution of time from the end of EVT to follow-up MRI.

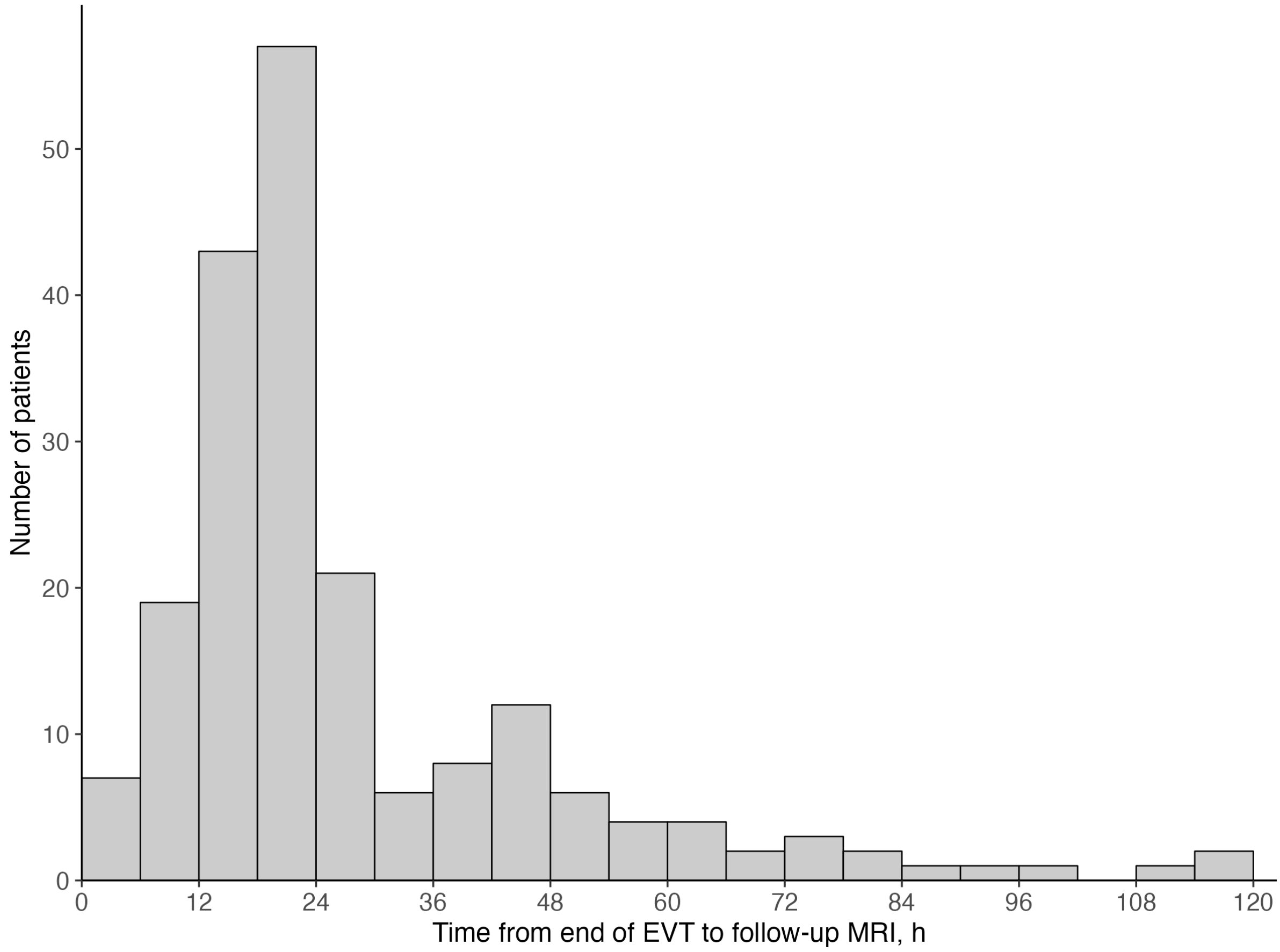

Abbreviations: EVT, endovascular thrombectomy; MRI, magnetic resonance imaging.

Table 1. Comparison of patient characteristics among included patients, patients without post-interventional FDCT, and other excluded patients.

	Included (n = 200)	No FDCT (n = 53)	Other excluded (n=62)	P-value
Age, y	78 [72–84]	79 [73–84]	79 [74–84]	0.62
Females	75 (37.5)	24 (45.3)	26 (41.9)	0.53
Premorbid mRS score	0 [0–2]	0 [0–0]	0 [0–1]	0.18
Baseline NIHSS score*	17 [11–23]	18 [14–24]	19 [15–25]	0.09
ASPECTS†	8 [6–9]	8 [7–10]	7 [5–9]	0.02
**Medical history**				
Hypertension	123 (61.5)	37 (69.8)	37 (59.7)	0.49
Diabetes mellitus	45 (22.5)	18 (34.0)	12 (19.4)	0.17
Dyslipidemia	81 (40.5)	15 (28.3)	19 (30.6)	0.16
Congestive heart failure	25 (12.5)	7 (13.2)	12 (19.4)	0.40
Atrial fibrillation	114 (57.0)	33 (62.3)	38 (61.3)	0.73
Previous stroke/TIA	51 (25.5)	13 (24.5)	13 (21.0)	0.79
Prior oral anticoagulants	33 (16.5)	14 (26.4)	17 (27.4)	0.08
Prior antiplatelets	53 (26.5)	7 (13.2)	12 (19.4)	0.10
**Initial site of occlusion**				
ICA	65 (32.5)	14 (26.4)	16 (25.8)	0.81
M1	78 (39.0)	22 (41.5)	30 (48.4)	
M2/M3	37 (18.5)	11 (20.8)	12 (19.4)	
Posterior circulation	20 (10.0)	6 (11.3)	4 (6.5)	
**First-line EVT strategy**				
SR	11 (5.5)	3 (5.7)	2 (3.2)	0.17

CA	107 (53.5)	24 (45.3)	23 (37.1)	
Combined SR and CA	79 (39.5)	24 (45.3)	36 (58.1)	
Other	3 (1.5)	2 (3.8)	1 (1.6)	
Intravenous thrombolysis	84 (42.0)	17 (32.1)	23 (37.1)	0.40
Number of thrombectomy passes	1 [1–3]	2 [1–2]	2 [1–2]	0.37
Onset-to-puncture time, min‡	264 [146–476]	251 [140–630]	268 [155–465]	0.91
Puncture-to-eTICI ≥2b reperfusion time, min	37 [24–61]	41 [25–59]	34 [25–52]	0.61

Data are presented as median [interquartile range] or number (%).

Categorical variables were compared using Fisher`s exact test, except for occlusion site, which was compared using Person's chi-squre test.

Abbreviations: FDCT, flat-panel detector computed tomography; mRS, modified Rankin Scale; NIHSS, National Institute of Health Stroke Scale; ASPECTS, Alberta Stroke Program Early CT score; TIA, transient ischemic attack; ICA, internal carotid artery; M1, first segment of the middle cerebral artery; M2, second segment of the middle cerebral artery; M3, third segment of the middle cerebral artery; EVT, endovascular thrombectomy; SR, stent retriever; CA, contact aspiration; eTICI, expanded Thrombolysis In Cerebral Infarction.

Table 2. Clinical and imaging characteristics of patients reclassified by FDCT-augmented TICI.

Case	Reclassification direction	Operator-graded TICI	FDCT-augmented TICI	Number of DOT signs	Baseline NIHSS score	ASPECTS	FIV, mL	HT	Discharge mRS
1	Downgraded	3	2b	1	8	10	4.79	No	1
2	Downgraded	2c	2b	2	16	4	116.11	HI2	4
3	Downgraded	2c	2b	3	15	8	54.50	PH1	5
4	Downgraded	2c	2b	2	23	6	105.51	HI2	5
5	Downgraded	2c	2b	0	12	10	3.71	No	2
6	Downgraded	2c	2b	1	33	10	214.23	HI2	5
7	Downgraded	3	2b	0	11	7	16.43	HI2	5
8	Downgraded	3	2b	0	25	8	83.43	No	6
9	Downgraded	2c	2b	1	6	8	2.71	No	4
10	Downgraded	2c	2b	1	15	10	149.80	HI1	5
11	Downgraded	2c	2b	1	6	8	3.71	HI1	0
12	Downgraded	2c	2b	0	21	8	86.21	PH1	5
13	Downgraded	2c	2b	4	24	7	170.11	No	4
14	Downgraded	3	2b	0	19	6	112.43	HI2	5
15	Upgraded	2b	3	0	36	6	37.88	No	4
16	Upgraded	2b	2c	0	15	5	110.13	HI2	5
17	Upgraded	2b	2c	0	29	4	84.58	No	5

Abbreviations: FDCT, flat-panel detector computed tomography; TICI, Thrombolysis In Cerebral Infarction; DOT, distal occlusion tracker; NIHSS, National Institute of Health Stroke Scale; ASPECTS, Alberta Stroke Program Early CT score; FIV, final infarct volume; HT, hemorrhagic transformation; mRS, modified Rankin Scale; HI, hemorrhagic infarction; PH, parenchymal hematoma.

Figure 3. Representative case classified as TICI 2c according to operator-graded TICI but as TICI 2b according to FDCT-augmented TICI.

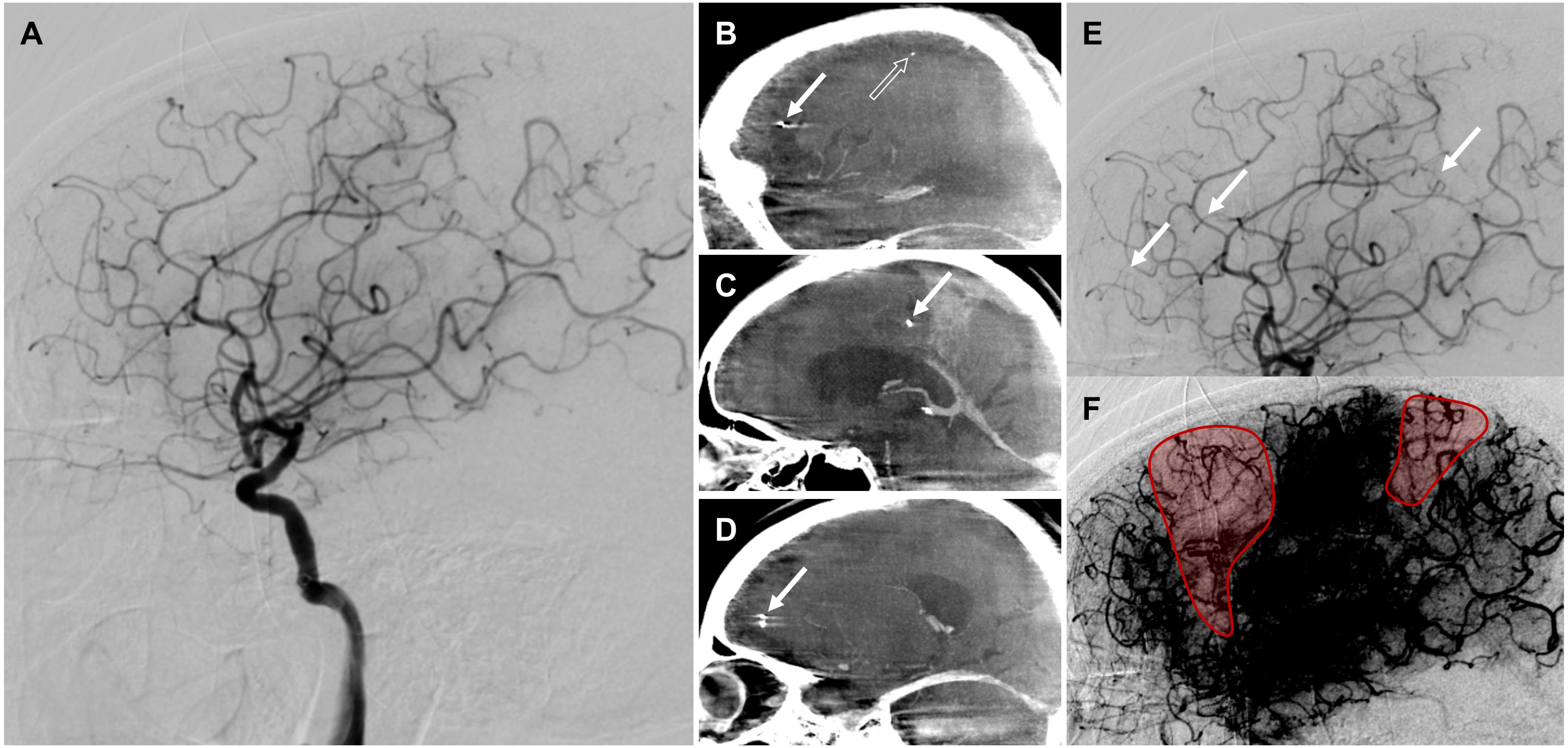

(A) A patient with left M1 distal occlusion was assessed as having near-complete reperfusion according to operator-graded TICI (grade 2c) at the end of the intervention. (B-D) Immediate post-interventional FDCT showing four positive DOT signs (*filled and open arrows*). (E, F) By correlating the final angiography with the DOT signs, residual vessel occlusions at the M4 level were identified at three of the four DOT-positive locations (*filled arrows*). Accordingly, the reperfusion status was graded as FDCT-augmented TICI 2b and classified as incomplete.

Abbreviations: TICI, Thrombolysis In Cerebral Infarction; FDCT, flat-panel detector computed tomography; M1, first segment of the middle cerebral artery; DOT, distal occlusion tracker; M4, fourth segment of the middle cerebral artery.

Figure 4. Association between TICI grades and DOT signs.

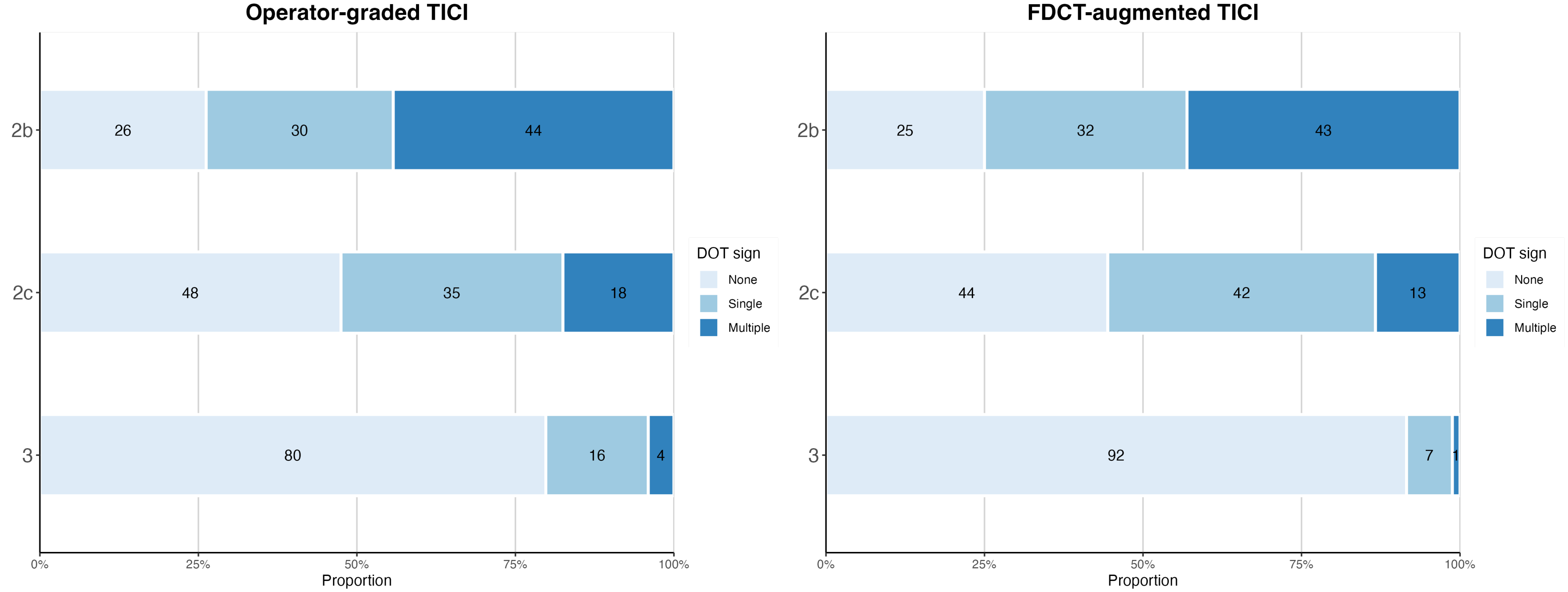

Abbreviations: TICI, Thrombolysis In Cerebral Infarction; DOT, distal occlusion tracker; FDCT, flat-panel detector computed tomography.

Table 3. Patient characteristics according to reperfusion status defined by FDCT-augmented TICI.

	Incomplete reperfusion (TICI 2b) (n = 72)	Near-complete/complete reperfusion (TICI 2c/3) (n = 128)	P-value
Age, y	77 [68–82]	79 [73–84]	0.23
Females	26 (36.1)	49 (38.3)	0.88
Premorbid mRS score	0 [0–1]	0 [0–2]	0.23
Baseline NIHSS score*	19 [12–23]	16 [10–24]	0.45
ASPECTS†	8 [6–9]	8 [6–9]	0.42
**Medical history**			
Hypertension	45 (62.5)	78 (60.9)	0.88
Diabetes mellitus	19 (26.4)	26 (20.3)	0.38
Dyslipidemia	24 (33.3)	57 (44.5)	0.14
Congestive heart failure	8 (11.1)	17 (13.3)	0.82
Atrial fibrillation	43 (59.7)	71 (55.5)	0.66
Previous stroke/TIA	15 (20.8)	36 (28.1)	0.31
Prior oral anticoagulants	14 (19.4)	19 (14.8)	0.43
Prior antiplatelets	15 (20.8)	38 (29.7)	0.19
**Initial site of occlusion**			
ICA	25 (34.7)	40 (31.2)	0.21
M1	29 (40.3)	49 (38.3)	
M2/M3	15 (20.8)	22 (17.2)	
Posterior circulation	3 (4.2)	17 (13.3)	
**First-line EVT strategy**			
SR	3 (4.2)	8 (6.2)	0.45
CA	43 (59.7)	64 (50.0)	
Combined SR and CA	26 (36.1)	53 (41.4)	
Other	0 (0.0)	3 (2.3)	
Intravenous thrombolysis	26 (36.1)	58 (45.3)	0.23
Number of thrombectomy passes	2 [1–3]	1 [1–2]	0.004
Onset-to-puncture time, min‡	304 [147–495]	255 [147–453]	0.51
Puncture-to-eTICI ≥2b reperfusion time, min	40 [25–61]	36 [23–61]	0.35

Time from end of EVT to FDCT, min	3 [2–7]	3 [2–9]	0.35
Time from end of EVT to follow-up MRI, h	22 [16–31]	21 [16–35]	0.58
**DOT sign**			
None	18 (25.0)	96 (75.0)	
Single	23 (31.9)	25 (19.5)	<0.001
Multiple	31 (43.1)	7 (5.5)	
Number of DOT signs	1 [1–3]	0 [0–0]	<0.001

Data are presented as median [interquartile range] or number (%).

*4 missing values (2 for incomplete reperfusion and 2 for near-complete reperfusion).

†ASPECTS was obtained using MRI (n = 88) or CT (n = 112).

‡9 missing values (5 for incomplete reperfusion and 4 for near-complete reperfusion).

Abbreviations: FDCT, flat-panel detector computed tomography; TICI, Thrombolysis In Cerebral Infarction; mRS, modified Rankin Scale; NIHSS, National Institute of Health Stroke Scale; ASPECTS, Alberta Stroke Program Early CT score; TIA, transient ischemic attack; ICA, internal carotid artery; M1, first segment of the middle cerebral artery; M2, second segment of the middle cerebral artery; M3, third segment of the middle cerebral artery; EVT, endovascular thrombectomy; SR, stent retriever; CA, contact aspiration; ; eTICI, expanded Thrombolysis In Cerebral Infarction; MRI, magnetic resonance imaging; DOT, distal occlusion tracker; CT, computed tomography.

Table 4. Patient characteristics according to reperfusion status defined by operator-graded TICI.

	Incomplete reperfusion (TICI 2b) (n = 61)	Near-complete/complete reperfusion (TICI 2c/3) (n = 139)	P-value
Age, y	77 [69–82]	79 [72–85]	0.21
Females	21 (34.4)	54 (38.8)	0.64
Premorbid mRS score	0 [0–1]	0 [0–2]	0.39
Baseline NIHSS score*	19 [13–23]	16 [10–23]	0.16
ASPECTS†	8 [6–9]	8 [7–9]	0.14
**Medical history**			
Hypertension	39 (63.9)	84 (60.4)	0.75
Diabetes mellitus	15 (24.6)	30 (21.6)	0.71
Dyslipidemia	23 (37.7)	58 (41.7)	0.64
Congestive heart failure	7 (11.5)	18 (12.9)	1.00
Atrial fibrillation	35 (57.4)	79 (56.8)	1.00
Previous stroke/TIA	16 (26.2)	35 (25.2)	0.86
Prior oral anticoagulants	12 (19.7)	21 (15.1)	0.42
Prior antiplatelets	14 (23.0)	39 (28.1)	0.49
**Initial site of occlusion**			
ICA	23 (37.7)	42 (30.2)	0.17
M1	25 (41.0)	53 (38.1)	
M2/M3	11 (18.0)	26 (18.7)	
Posterior circulation	2 (3.3)	18 (12.9)	
**First-line EVT strategy**			
SR	3 (4.9)	8 (5.8)	0.35
CA	38 (62.3)	69 (49.6)	
Combined SR and CA	20 (32.8)	59 (42.4)	
Other	0 (0.0)	3 (2.2)	
Intravenous thrombolysis	24 (39.3)	60 (43.2)	0.64
Number of thrombectomy passes	2 [1–3]	1 [1–2]	0.001
Onset-to-puncture time, min‡	282 [149–471]	256 [145–475]	0.62
Puncture-to-eTICI ≥2b reperfusion time, min	43 [28–62]	35 [23–61]	0.09

Time from end of EVT to FDCT, min	3 [2–7]	3 [2–9]	0.67
Time from end of EVT to follow-up MRI, h	22 [17–38]	21 [15–30]	0.59
**DOT sign**			
None	16 (26.2)	98 (70.5)	
Single	18 (29.5)	30 (21.6)	<0.001
Multiple	27 (44.3)	11 (7.9)	
Number of DOT signs	1 [0–3]	0 [0–1]	<0.001

Data are presented as medians [interquartile ranges] or numbers (%).

*4 missing values (2 for incomplete reperfusion and 2 for near-complete reperfusion).

†ASPECTS was obtained using MRI (n = 88) or CT (n = 112).

‡9 missing values (4 for incomplete reperfusion and 5 for near-complete reperfusion).

Abbreviations: FDCT, flat-panel detector computed tomography; TICI, Thrombolysis In Cerebral Infarction; mRS, modified Rankin Scale; NIHSS, National Institute of Health Stroke Scale; ASPECTS, Alberta Stroke Program Early CT score; TIA, transient ischemic attack; ICA, internal carotid artery; M1, first segment of the middle cerebral artery; M2, second segment of the middle cerebral artery; M3, third segment of the middle cerebral artery; EVT, endovascular thrombectomy; SR, stent retriever; CA, contact aspiration; ; eTICI, expanded Thrombolysis In Cerebral Infarction; MRI, magnetic resonance imaging; DOT, distal occlusion tracker; CT, computed tomography.

Figure 5. Association between DOT signs and final infarct volume stratified by TICI grade.

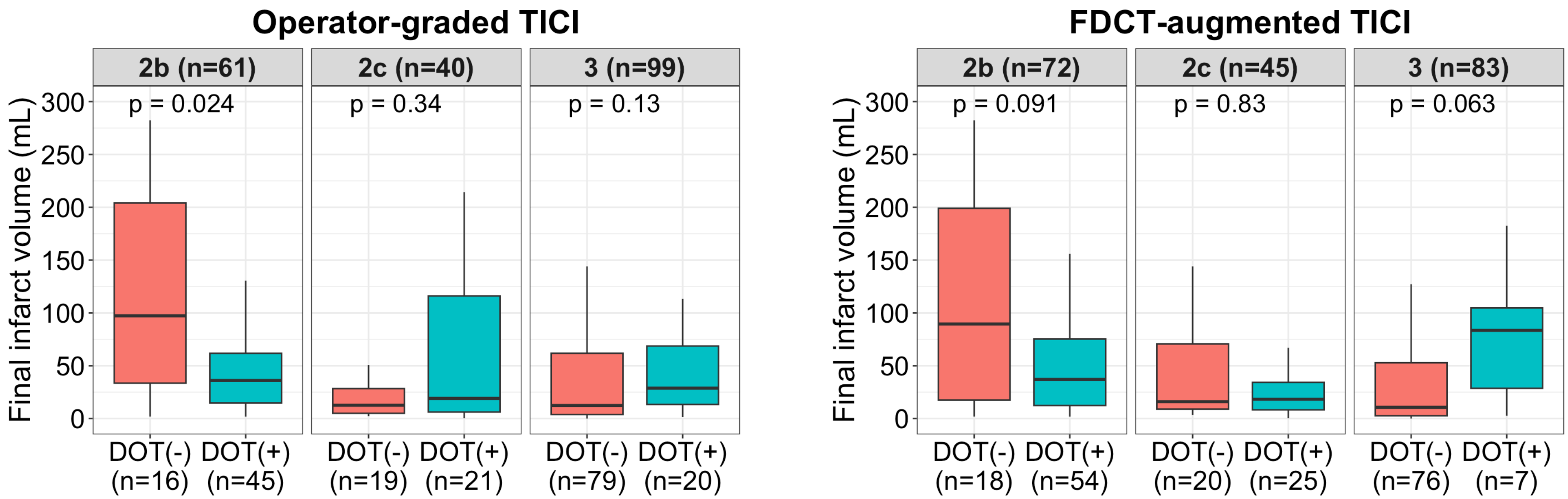

Abbreviations: DOT, distal occlusion tracker; TICI, Thrombolysis In Cerebral Infarction; FDCT, flat-panel detector computed tomography.

Figure 6. Final infarct volume stratified by the presence of DOT signs according to reclassification by FDCT-augmented TICI.

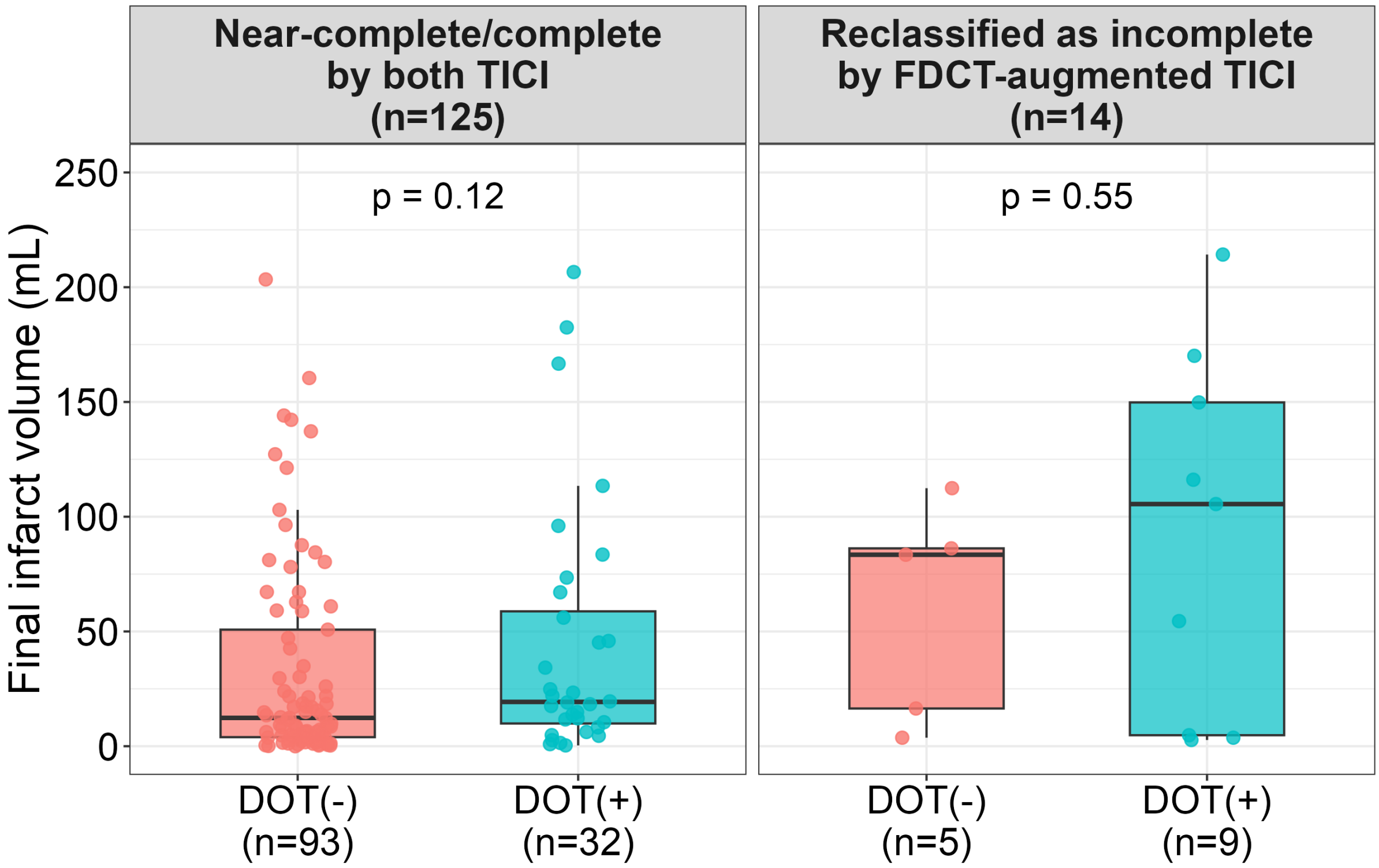

Abbreviations: DOT, distal occlusion tracker; FDCT, flat-panel detector computed tomography; TICI, Thrombolysis In Cerebral Infarction.

Table 5. Model performance of multivariable linear regression models for final infarct volume among patients who underwent follow-up MRI within 48 hours after EVT.

	Operator-graded TICI	FDCT-augmented TICI
Incomplete reperfusion (TICI 2b), β coefficient (95% CI)	11.8 (−12.2–35.9)	22.5 (0.3–44.8)
**Model performance**		
Adjusted R^2	0.265	0.279
AIC	1923.0	1919.8
BIC	1963.8	1960.6
**Model difference favoring FDCT-augmented TICI**		
ΔAdjusted R^2	0.014 (-0.003–0.050)	
ΔAIC	3.229 (-0.805–12.164)	
ΔBIC	3.229 (-0.805–12.164)	

Adjusted for age, sex, baseline NIHSS score, ASPECTS, occlusion site, time from puncture to reperfusion to eTICI ≥2b, the number of passes, and time from the end of EVT to follow-up MRI.

Abbreviations: MRI, magnetic resonance imaging; EVT, endovascular thrombectomy; TICI, Thrombolysis In Cerebral Infarction; FDCT, flat-panel detector computed tomography; CI, confidence interval; AIC, Akaike Information Criterion; BIC, Bayesian Information Criterion; NIHSS, National Institute of Health Stroke Scale; ASPECTS, Alberta Stroke Program Early CT score; eTICI, expanded Thrombolysis In Cerebral Infarction.

Figure 7. Forest plot of unstandardized regression coefficients for predictors of final infarct volume in models using two definitions of incomplete reperfusion.

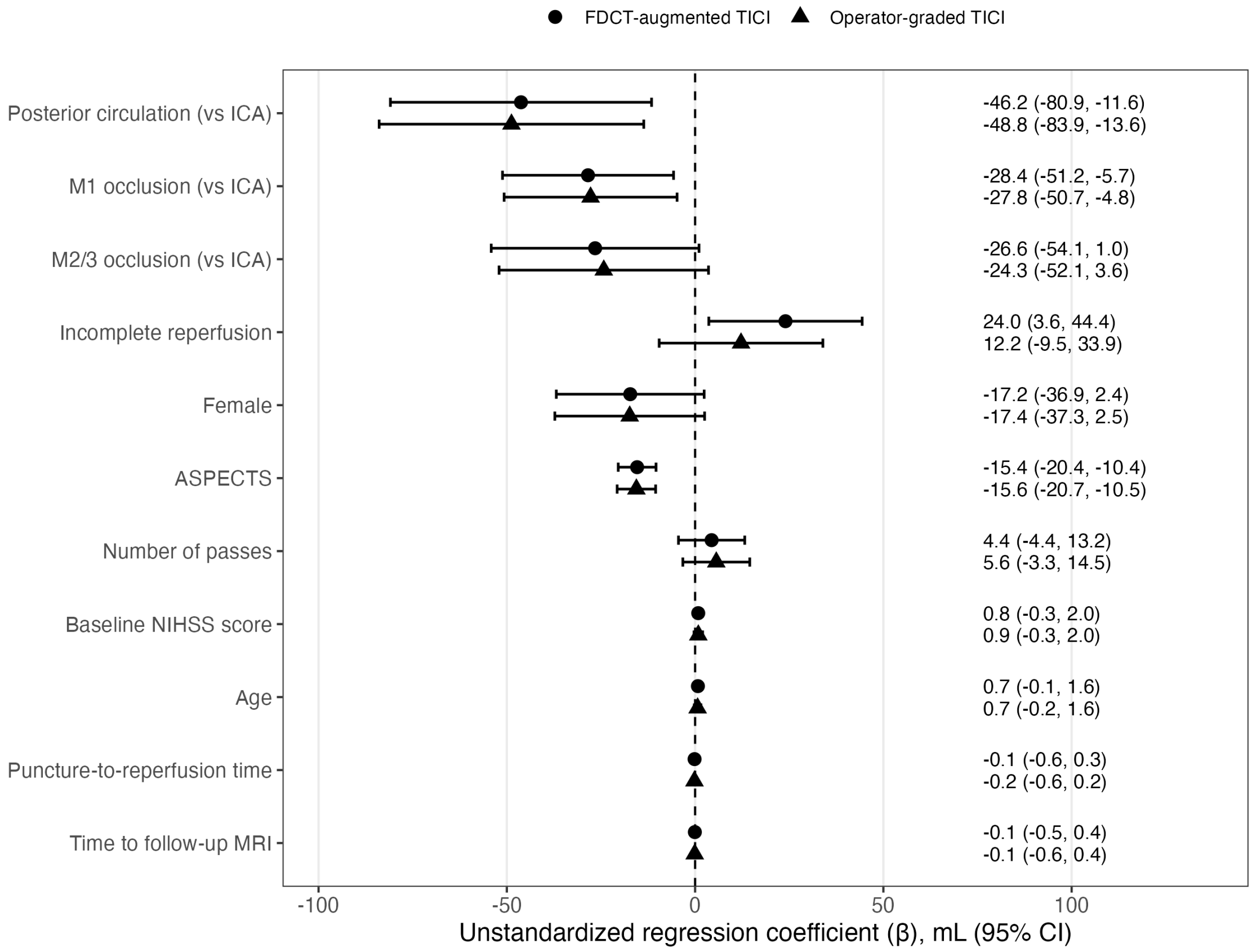

Abbreviations: FDCT, flat-panel detector computed tomography; TICI, Thrombolysis In Cerebral Infarction; ICA, internal carotid artery; M1, first segment of the middle cerebral artery; M2/3, second or third segment of the middle cerebral artery; ASPECTS, Alberta Stroke Program Early CT score; NIHSS, National Institute of Health Stroke Scale; MRI, magnetic resonance imaging.

Figure 8. Standardized regression coefficients for predictors of final infarct volume using two definitions of incomplete reperfusion.

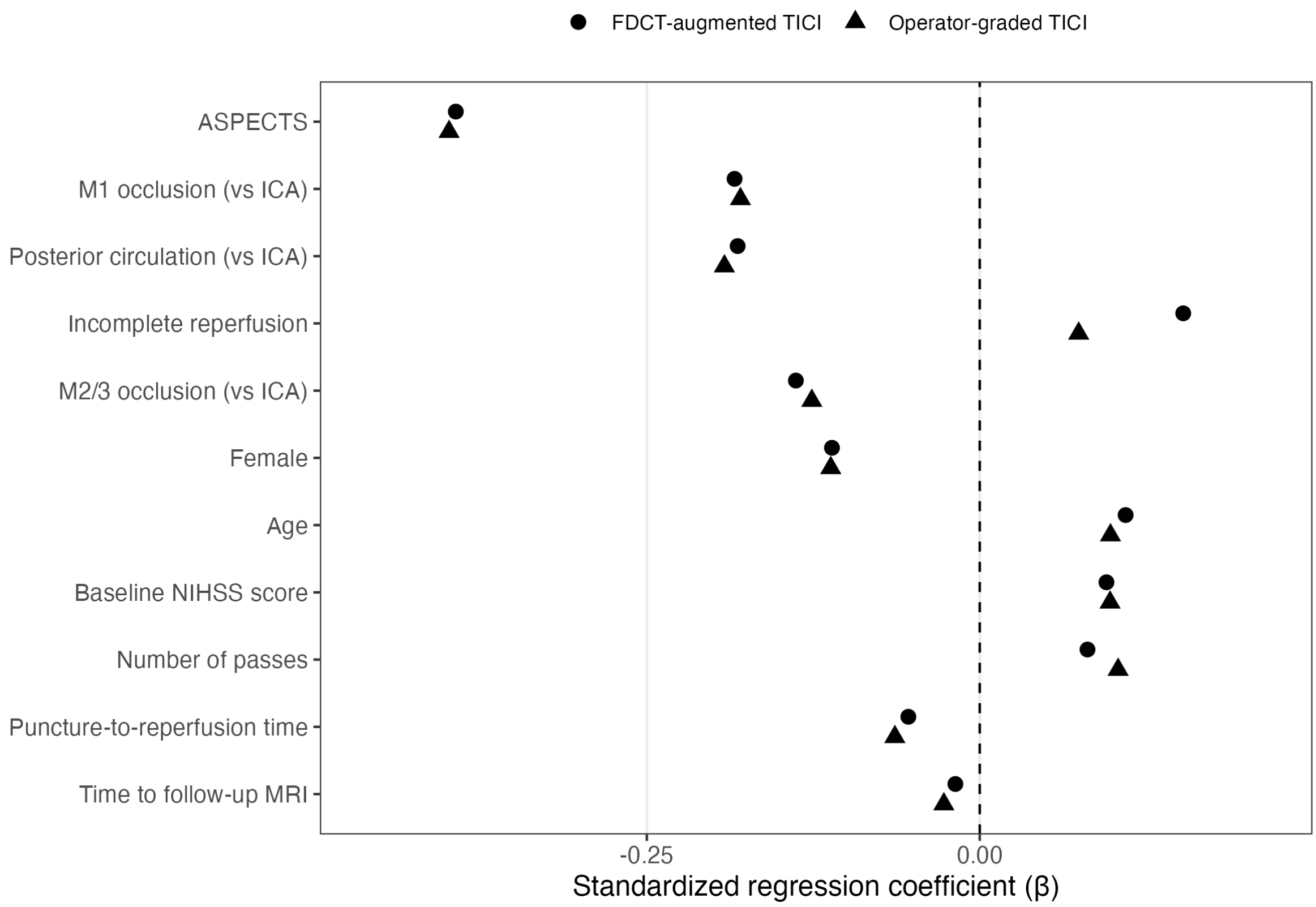

Abbreviations: FDCT, flat-panel detector computed tomography; TICI, Thrombolysis In Cerebral Infarction; ASPECTS, Alberta Stroke Program Early CT score; M1, first segment of the middle cerebral artery; ICA, internal carotid artery; M2/3, second or third segment of the middle cerebral artery; NIHSS, National Institute of Health Stroke Scale; MRI, magnetic resonance imaging.

Table 6 Outcomes according to reperfusion status defined by FDCT-augmented TICI.

	Incomplete reperfusion (TICI 2b) (n = 72)	Near-complete/complete reperfusion (TICI 2c/3) (n = 128)	Unadjusted OR (95% CI)	Adjusted OR (95% CI)
**Safety outcomes***				
Any hemorrhagic transformation	28 (38.9)	35 (27.3)	1.69 (0.91–3.12)	1.68 (0.85–3.32)
Hemorrhagic infarction	21 (29.2)	33 (25.8)	1.19 (0.62–2.25)	1.22 (0.60–2.47)
Parenchymal hematoma	7 (9.7)	2 (1.6)	6.78 (1.59–46.42)	5.16 (0.97–27.33)
Symptomatic ICH	3 (4.2)	3 (2.3)	1.81 (0.33–10.02)	2.87 (0.28–29.53)
Subarachnoid hemorrhage	13 (18.1)	6 (4.7)	4.48 (1.68–13.30)	4.05 (1.31–12.51)
**Clinical outcomes†**				
Favorable outcome	15 (20.8)	46 (35.9)	0.47 (0.23–0.90)	0.35 (0.15–0.80)
Excellent outcome	10 (13.9)	34 (26.6)	0.45 (0.20–0.94)	0.40 (0.16–0.99)
mRS score shift analysis	–	–	–	0.61 (0.34–1.06)

Data are presented as numbers (%).

*Models were adjusted for age, sex, baseline NIHSS score, ASPECTS, occlusion site, intravenous thrombolysis, and the number of passes.

†Models were adjusted for age, sex, baseline NIHSS score, ASPECTS, occlusion site, time from puncture to reperfusion to eTICI ≥2b, and the number of passes.

Abbreviations: FDCT, flat-panel detector computed tomography; TICI, Thrombolysis In Cerebral Infarction; OR, odds ratio; CI, confidence interval; ICH, intracranial hemorrhage; mRS, modified Rankin Scale; NIHSS, National Institute of Health Stroke Scale; ASPECTS, Alberta Stroke

Program Early CT score, eTICI, expanded Thrombolysis In Cerebral Infarction.

Table 7. Outcomes according to reperfusion status defined by operator-graded TICI.

	Incomplete reperfusion (TICI 2b) (n = 61)	Near-complete/complete reperfusion (TICI 2c/3) (n = 139)	Unadjusted OR (95% CI)	Adjusted OR (95% CI)
**Safety outcomes***				
Any hemorrhagic transformation	20 (32.8)	43 (30.9)	1.09 (0.57–2.06)	0.91 (0.44–1.88)
Hemorrhagic infarction	15 (24.6)	39 (28.1)	0.84 (0.41–1.64)	0.74 (0.34–1.61)
Parenchymal hematoma	5 (8.2)	4 (2.9)	3.01 (0.77–12.57)	2.04 (0.48–8.71)
Symptomatic ICH	3 (4.9)	3 (2.2)	2.34 (0.42–13.00)	3.80 (0.39–37.33)
Subarachnoid hemorrhage	11 (18.0)	8 (5.8)	3.60 (1.38–9.81)	3.25 (1.09–9.65)
**Clinical outcomes†**				
Favorable outcome	12 (19.7)	49 (35.3)	0.45 (0.38–0.90)	0.38 (0.16–0.93)
Excellent outcome	8 (13.1)	36 (25.9)	0.43 (0.18–0.95)	0.45 (0.17–1.21)
mRS score shift analysis	–	–	–	0.78 (0.43–1.40)

Data are presented as numbers (%).

*Models were adjusted for age, sex, baseline NIHSS score, ASPECTS, occlusion site, intravenous thrombolysis, and the number of passes.

†Models were adjusted for age, sex, baseline NIHSS score, ASPECTS, occlusion site, time from puncture to reperfusion to eTICI ≥2b, and the number of passes.

Abbreviations: TICI, Thrombolysis In Cerebral Infarction; OR, odds ratio; CI, confidence interval; ICH, intracranial hemorrhage; mRS, modified Rankin Scale; NIHSS, National Institute of Health Stroke Scale; ASPECTS, Alberta Stroke Program Early CT score, eTICI, expanded

Thrombolysis In Cerebral Infarction.
